# Analysis of post-COVID symptoms and predisposing factors for chronic post-COVID syndrome

**DOI:** 10.5578/tt.20239606

**Published:** 2023-12-11

**Authors:** Hülya ABALI, Dilara DEMİR, Şule GÜL, Nurdan ŞİMŞEK VESKE, Seda TURAL ONUR

**Affiliations:** 1 Clinic of Pulmonology, Yedikule Chest Diseases and Thoracic Surgery Training and Research Hospital, University of Health Sciences, İstanbul, Türkiye; 2 Clinical Psychology, Neurovia Neuropsychiatry Clinic, İstanbul, Türkiye

## Abstract

**ABSTRACT**

**
Analysis of post-COVID symptoms and predisposing factors for chronic post-COVID syndrome
**

**Introduction:** *While there is sufficient information about acute COVID-19, which can cause a multisystemic and fatal disease, post-COVID syndrome and risk factors for this condition remain poorly known. We aimed to identify post- COVID symptoms and risk factors for chronic post-COVID syndrome through this study.
*

**Materials and Methods:** *This prospective cross-sectional study was conducted on 254 out of 384 COVID-19 patients admitted to our COVID-19 polyclinic between February and April 2021. The patients were questioned with a list of 37 symptoms at the fifth and twelfth weeks after disease onset via phone review, and their acute post-COVID (APC) and chronic post-COVID (CPC) symptoms were recorded. Data on risk factors were collected from the hospi- tal’s medical records system. Associations between symptom count in the CPC phase and age, sex, hospitalization, RT-PCR result, specific radiological find- ings, comorbidities, and long-term medications were evaluated.
*

**Results:** *Two hundred twenty-one patients had APC symptoms, and 138 patients had CPC symptoms. While the most common symptom was fatigue at week five, it was hair loss at week 12. Symptoms were observed signifi- cantly less in the CPC phase than in the APC phase (Z= -12.301, p= 0.00). Female sex and the presence of specific radiological findings were signifi- cantly associated with the occurrence of CPC symptoms (p= 0.03, p= 0.00, respectively). Long-term use of angiotensin-2 receptor blockers (ARBs) was correlated with a low symptom count in the CPC phase (p= 0.00).
*

**Conclusion:** *Female sex and the presence of specific radiological findings were risk factors for developing CPC. Long-term use of ARBs was associated with a low chronic post-COVID symptom burden. A substantial cluster of multisys- temic symptoms was observed in both phases, and this condition highlights the requirement for customized outpatient management that includes long-term follow-up and treatment of COVID-19 patients. Identifying the high-risk patients that will develop persistent symptoms can guide this management.
*

**Key words:** *Chronic post-COVID; symptoms; risk factors; female; ACE2 receptor blockers
*

**ÖZ**

**
Post-COVID semptomlarının analizi ve kronik post-COVID sendromu için predispozan faktörler
**

**Giriş:** *Multisistemik ve ölümcül bir hastalığa neden olabilen akut COVID-19 hakkında yeterli bilgi bulunurken, post-COVID sendromu ve bu durum için risk faktörleri halen çok az bilinmektedir. Bu çalışma ile post-COVID semptomlarını ve kronik post-COVID sendro- mu için risk faktörlerini belirlemeyi amaçladık.
*

**Materyal ve Metod:** *Bu prospektif kesitsel çalışma, Şubat-Nisan 2021 tarihleri arasında COVID-19 polikliniğimize başvuran 384 COVID- 19 hastasının 254’ü üzerinde gerçekleştirildi. Hastalar, hastalığın başlangıcından sonraki beşinci ve 12. haftalarda 37 semptomdan oluşan bir liste ile telefonla sorgulandı ve bu hastaların akut post-COVID (APC) ve kronik post-COVID (KPC) semptomları kaydedildi. Risk faktörlerine ilişkin veriler hastanenin medikal kayıt sisteminden toplandı. KPC aşamasındaki semptom sayısı ile yaş, cinsiyet, hastaneye yatış, RT-PCR sonucu, spesifik radyolojik bulgular, komorbiditeler ve uzun süreli ilaç kullanımı arasındaki ilişkiler değerlendirildi.
*

**Bulgular:** *İki yüz elli dört hastadan 221’inde APC semptomlar ve 138 hastada KPC semptomlar mevcuttu. En sık görülen semptom beşinci haftada yorgunlukken, 12. haftada saç dökülmesiydi. Semptomlar KPC fazında APC fazına göre anlamlı olarak daha az göz- lendi (Z= -12,301, p= 0,00). Kadın cinsiyet ve spesifik radyolojik bulguların varlığı, KPC semptomlarının ortaya çıkmasıyla anlamlı düzeyde ilişkiliydi (sırasıyla p= 0,03, p= 0,00). Anjiyotensin-2 reseptör blokerlerinin (ARB’ler) uzun süreli kullanımı, KPC fazındaki düşük semptom sayısıyla ilişkiliydi (p= 0,00).
*

**Sonuç:** *Kadın cinsiyet ve spesifik radyolojik bulguların varlığı, KPC gelişimi için risk faktörleriydi. ARB’lerin uzun süreli kullanımı, düşük KPC semptom yüküyle ilişkilendirildi. Her iki fazda da önemli multisistemik semptom kümesi gözlemlendi ve bu durum, COVID-19 hastalarının uzun süreli takibini ve tedavisini içeren özelleştirilmiş bir ayakta tedavi yönetimi gerekliliğini vurgulamaktadır. Kalıcı semp- tomlar geliştirecek yüksek riskli popülasyonun belirlenmesi bu yönetime yol gösterebilir.
*

**Anahtar kelimeler:** *Kronik post-COVID; semptomlar; risk faktörleri; kadın cinsiyet; ACE2 reseptör blokerleri
*

## INTRODUCTION

The Coronavirus disease-2019 (COVID-19), which caused approximately 6 million 400 thousand deaths as of July 2022, has been a health, economic, and social burden worldwide since its outbreak in Wuhan, China, in December 2019 (1). The etiopathogenesis, clinical manifestations, and course of patients with COVID-19 during the acute period have been clearly described. However, post-COVID syndrome remains primarily uncertain (2).

Long COVID is defined as any post-COVID symptom present following the SARS-CoV-2 infection, and it has two consecutive stages: acute post-COVID (between five and 12 weeks after the onset of symptoms) and chronic post-COVID (persistent longer than 12 weeks) (3).

The incidence of post-COVID syndrome is 43% globally, 51% in Asia, 44% in Europe, and 31% in the USA. Post-COVID syndrome develops in 54% of hospitalized patients and 34% of non-hospitalized patients (4). Healthcare professionals, social care providers, and policymakers should inform the community about this condition, which is notably common, and reduce anxiety (5). Predicting persistent symptoms plays a crucial role in avoiding future, potentially more catastrophic health issues. It is, therefore, crucial to identify predictors of broad- ranged symptoms to chart a roadmap for managing this condition effectively.

Chronic post-COVID syndrome (CPCS) has a better clinical course compared to acute COVID-19 infection.

In patients who develop CPCS, acute COVID-19 symptoms gradually decrease, although the specific predictors for CPCS development are still insufficiently defined (6). In this study, we aimed to identify the post- COVID symptoms and the predisposing factors for CPCS in COVID-19 patients. Based on the study results, clinicians can promptly refer high-risk patients, who are likely to develop CPCS, to the appropriate health and social care providers.

## MATERIALS and METHODS

**Study Design and Setting**

This single-center, prospective cross-sectional study was performed on the patients diagnosed with COVID-19 by positive real-time polymerase chain reaction (RT-PCR) testing and/or specific radiological findings following the World Health Organization (WHO) guidelines.

A randomized sample of 384 COVID-19 patients (1:5 ratio) who had visited the COVID-19 polyclinic at the reference chest diseases center between February and April 2021 was selected. A flowchart of the patient recruitment into the study is shown in Figure 1. Out of 384 patients, 254 patients agreed to participate in the study, and they were probed with a list of 37 symptoms at weeks five and twelve after disease onset. The APC and CPC symptoms that patients declared were recorded. The symptom count in the CPC phase was compared to the following variables: age, sex, hospitalization, RT-PCR result, specific radiological findings, comorbidities, and long-term medications.

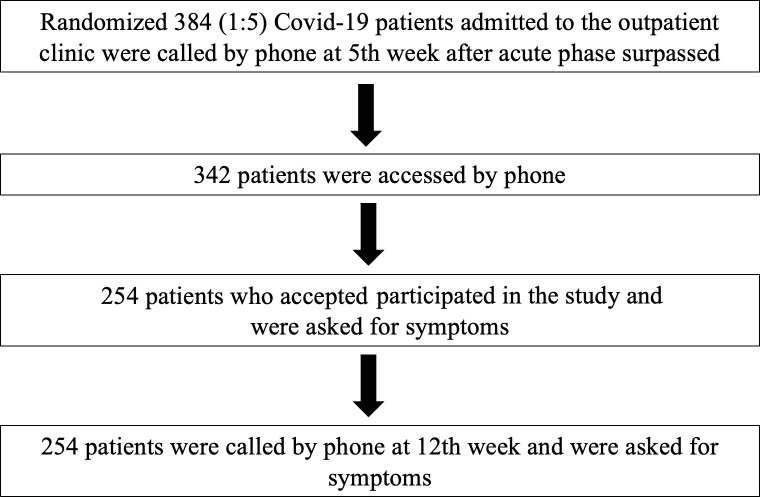

**Figure 1.** Flowchart of the patient recruitment.

## Ethical Consideration

The study was performed following Good Clinical Practices and the Declaration of Helsinki. The study was approved by İstanbul Yedikule Chest Diseases and Thoracic Surgery Training and Research Hospital Clinical Research Ethics Committee (Date: 06.05.2021, Approval no: 2021-119) and informed consent was obtained from all participants.

## Data Collection

Data on demographic characteristics (age, sex), site of initial hospitalization (none, clinic, intensive care unit), RT-PCR testing result, presence of specific radiological findings on thoracic CT, pre-existing comorbidities [chronic pulmonary diseases, chronic liver diseases, chronic renal diseases, cardiovascular diseases, hypertension (HT), diabetes mellitus (DM), malignancy, immunodeficiency, collagen vascular diseases, cerebrovascular diseases], and long-term medications (acetylsalicylic acids, proton pump inhibitors, analgesics, anticoagulants, antihyperten- sives, antidiabetics, antirheumatic drugs, antiar- rhythmics, antidepressants, bronchodilators, antiepi- leptics, cholesterol drugs, and others) were recorded from the hospital’s electronic registration system.

Information on symptoms was collected via phone interview. The patients were questioned for a group of symptoms (cough, fever, exertion dyspnea, chest pain, sputum, myalgia, body pain, joint pain, fatigue, sweating, weight gain, weight loss, hair loss, sore throat, runny nose, dry throat, postnasal drip, nasal congestion, loss of smell, loss of taste, paresthesia in

hands and feet, headache, dizziness, impairments of concentration, amnesia, insomnia, unhappiness, anxiety, palpitation, blurred vision, dry eye, eye flashings, ear congestion, tinnitus, abdominal pain, diarrhea, nausea-vomiting) in the APC and CPC phases, respectively.

## Definitions

While acute post-COVID symptoms were defined as symptoms that persisted at week five after the disease onset, chronic post-COVID symptoms were defined as symptoms that persisted at week 12 after the disease onset. Specific radiological findings were described as bilateral and peripheral ground glass opacities and consolidation based on WHO guidelines. Initial hospitalization was defined as hospitalization within five weeks of disease onset.

## Statistical Analysis

IBM SPSS Statistics 24.00 software was used to perform statistical analyses. While the Mann- Whitney U test was used to compare the symptom count at the twelfth week based on sex, result of RT-PCR testing, presence of specific radiological findings, and comorbidities, the Kruskal-Wallis H test was used to compare the symptom count based on age and initial hospitalization. For post hoc analysis, the Mann-Whitney U test was used. A two- way analysis of variance was applied to compare the symptom count at the fifth and twelfth weeks according to the long-term medications used by patients. A value of p< 0.05 was deemed significant.

## RESULTS

Of the total 254 patients, 53.9% were female, and the mean age was 49.28. While RT-PCR testing was found positive in 91.3% of total patients, specific radiological findings were observed in 37% of total

patients. RT-PCR tests were positive in 72 (77%) and negative in 22 (23%) patients with specific radiological findings. Information on symptom count, initial hospitalization, and demographic and diagnostic characteristics of the patients was presented in Table 1.

**Table:** 

**Table 1.** Information on symptom count, initial hospitalization, demographic and diagnostic characteristics of COVID-19 patients	
**Total n= 254**	**n %**
**Gender**	
Female	137 53.9
Male	117 46.1
**Age, mean ± SD**	49.28 ± 15.47
Young (18-35 yrs)	55 21.7
Middle-aged (36-64 yrs)	156 61.4
Elderly (≥65 yrs)	43 16.9
**RT-PCR testing result**	
Negative	22 8.7
Positive	232 91.3
**Specific radiological findings**	94 37.0
**Comorbidities, one or more**	
Chronic pulmonary diseases	44 17.3
Chronic liver diseases	2 0.8
Chronic renal diseases	3 1.2
Cardiovascular diseases	31 12.2
Hypertension	80 31.5
Diabetes	43 16.9
Malignancy	10 3.9
Immunodeficiency	1 0.4
Collagen vascular disease	3 1.2
Cerebrovascular disease	3 1.2
**Initial hospitalization §**	
No	179 70.5
Clinic	59 23.2
Intensive care unit	16 6.3
**Mean symptom count of APC phase ± SD**	10.20 ± 7.73
None	13 5.1
1-2	23 9.1
≥3	218 85.8
**Mean symptom count of CPC phase ± SD**	2.78 ± 4.37
None	71 28.0
1-2	84 33.0
≥3	99 39.0
n: Number of patients, %: Percentage of patients, RT-PCR: Real-time polymerase chain reaction, APC: Acute post-COVID, CPC: Chronic post-COVID, SD: Standard deviation. §Hospitalization within five weeks after the COVID-19 disease onset.

Symptoms were observed in 221 (87%) patients in the acute post-COVID (APC) phase, and the five most common symptoms were the following; fatigue (67.9%), musculoskeletal pain (62.9%), cough (62.4%), joint pain (55.7%), and exertion dyspnea (55.2%). Symptoms were observed in 138 (54.3%) patients in the chronic post-COVID (CPC) phase,

and the five most common symptoms were as follows: hair loss (34.8%), weight gain (31.9%), joint pain (29.7%), fatigue (29%), and exertion dyspnea (28.3%) (Figure 2). Patients were more symptomatic in the APC phase than in the CPC phase (n= 221 vs. 138).

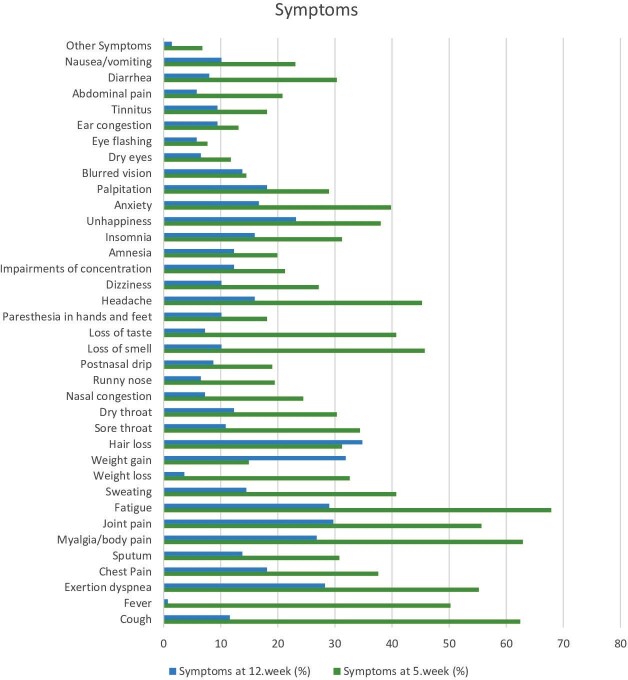

**Figure 2.** Incidence of acute and chronic post-COVID symptoms.

The Wilcoxon signed ranks test revealed that the symptom count in the acute post-COVID syndrome (APCS) was higher than the symptom count in the CPCS (Z= -12.301, p= 0.00). While the mean standard deviation value of the symptom count was

2. ± 7.7 in the APC phase, this value was 2.8 ± 4.4 in the CPC phase.

There was no significant correlation between symptom count in the CPC phase and age (p= 0.16). No statistically significant difference appeared between the symptom count in the CPC phase of the patients who were not hospitalized and the patients who were hospitalized (p= 0.16) (Table 2).

A significant association was observed between sex and the symptom count in the CPC phase, and the symptom count appeared higher in females compared to males (p= 0.03). While a significant correlation was not found between the RT-PCR testing results and the symptom count in the CPC phase (p= 0.69), a significant correlation was found between specific radiological findings and the symptom count in the CPC phase. The symptom count was higher in patients with specific radiological findings compared with the patients without specific radiological findings (p= 0.00) (Table 2).

The five most commonly used long-term medications were as follows, in order of frequency: antihypertensives, analgesics, proton pump inhibitors, antidiabetics, and bronchodilators (Table 3). Among long-term medications, only the use of ARBs was correlated with a low symptom count in the CPC phase (p= 0.00) (Table 4).

## DISCUSSION

Even months after the acute SARS-CoV-2 infection, patients are being admitted to the hospital with various morbidities that do not necessitate hospitalization, even if PCR testing is negative (7). There is still insufficient information about this condition, which is called “long COVID” or “post- COVID syndrome.” We aimed to contribute to the limited studies on APCS, CPCS, and predictors of CPCS with the outcomes of our study.

Patients participating in this study were questioned with a list of 37 symptoms, and it was observed that the patients had a broad spectrum of acute and chronic post-COVID symptoms. Several studies have

declared that fatigue is the most common symptom of the post-COVID syndrome (8-10). Similarly, fatigue, myalgia/body pain, cough, and joint pain were found to be the most common symptoms in APCS, in order of frequency. However, hair loss, weight gain, joint pain, and fatigue were the most common symptoms of CPCS, respectively, in this study. Telogen effluvium (TE), which appears with diffuse hair shedding 2-3 months after a triggering factor, is the most frequent cause of non-scarring alopecia (11). TE has been shown to manifest between two and 12 weeks after acute COVID-19 infection (12,13). A Turkish study demonstrated that COVID-19-associated TE (CATE) was experienced in 27.9% of 204 patients on a mean of 53.8 (± 23.8) days after COVID-19 PCR positivity (14). In line with the above-mentioned studies, CATE was observed in 31.2% of 254 patients in the twelfth week after acute COVID-19 infection in the present study. While the least apparent symptom was eye flashings (7.7%) in APCS, the least apparent symptom was fever (0.7%) in CPCS.

Similar to a systematic review, the symptom count in the CPCS was found to be lower compared to the symptom count in the APCS (15). Moreover, acute infection symptoms such as fever, weight loss, fatigue, sweating, and myalgia/body pain had markedly improved in CPCS. However, only the count of weight gain among the symptoms was observed to be increased in CPCS. This outcome might be attributed to the decrease in physical activity, malnutrition due to social isolation, and increased depressive symptoms in patients during the post-COVID period (16-18). Another reason for weight gain in patients might be hyperphagia due to post-traumatic stress disorder (19). The fact that COVID-19 disease has such a broad range of persistent symptoms in different organ systems indicates that SARS-CoV-2 causes a multi-systemic infection and chronic complications.

A study showed that older age (>50) was a predictor for symptom persistence, and an age between 40-49 years was found to be a predictor for CPCS in another study (20,21). Similar to a previous study, there was no correlation between age and the persistence of post-COVID symptoms in this study (22). Different study populations, ethnicity, symptom questioning time points, and forms of inquiry (e.g., questionnaire, phone review, face-to-face) may lead to this heterogeneity of the results related to age.

**Table:** 

**Table 2.** Association of the symptom count in the chronic post-COVID phase with age, initial hospitalization, gender, RT-PCR testing results, specific radiological findings, and comorbidities							
** Symptom count in chronic post-COVID phase **							
**n Mean SD Mean rank ϰ2 p**							
**Age**							
Young (18-35 yrs)		55	1.80	3.31	112.9	3.606	0.16
Middle-aged (36-64 yrs)		156	3.17	4.73	133.5		
Elderly (≥65 yrs)		43	2.58	4.07	124.6		
Total		254	2.78	4.37			
**Initial hospitalization §**							
No		179	2.51	4.29	122.1	3.669	0.16
Clinic		59	3.31	4.59	140.0		
Intensive care unit		16	3.75	4.34	141.9		
Total		254	2.78	4.37			
						**Z**	**p**
**Gender**							
Female		137	3.42	4.95	136.3		
Male		117	2.03	3.45	117.2	-2.187	0.03*
Total		254	2.78	4.37			
**RT-PCR testing result**							
Negative		22	2.55	3.57	133.2		
Positive		232	2.80	4.44	126.9	-0.402	0.69
Total		254	2.78	4.37			
**Specific radiological findings**							
No		160	1.95	3.76	109.7		
Yes		94	4.18	4.96	157.9	-5.325	0.00*
Total		254	2.78	4.37			
**Comorbidities**							
Chronic pulmonary diseases	No	210	2.70	4.34	126.8	-0.339	0.73
	Yes	44	3.11	4.53	130.7		
Chronic liver diseases	No	252	2.77	4.38	127.5	-0.127	0.90
	Yes	2	3.00	4.24	133.8		
Chronic renal diseases	No	251	2.78	4.39	127.4	-0.175	0.86
	Yes	3	2.33	3.22	134.5		
Cardiovascular diseases	No	223	2.83	4.43	127.5	-0.029	0.98
	Yes	31	2.39	3.93	127.8		
Hypertension	No	174	2.41	3.77	124.6	-0.963	0.34
	Yes	80	3.58	5.40	133.7		
Diabetes	No	211	2.67	4.21	126.2	-0.656	0.51
	Yes	43	3.30	5.10	133.9		
Malignancy	No	244	2.84	4.44	128.0	-0.567	0.57
	Yes	10	1.10	1.37	115.3		
Immunodeficiency	No	253	2.77	4.38	127.2	-1.056	0.29
	Yes	1	5.00	.	201.0		
Collagen vascular diseases	No	251	2.77	4.39	127.1	-0.887	0.37
	Yes	3	3.00	3.46	163.0		
Cerebrovascular disease	No	251	2.81	4.39	128.3	-1.725	0.08
	Yes	3	0.00	0.00	58.5		
n: Number of patients, SD: Standard deviation, RT-PCR: Real-time polymerase chain reaction. §Hospitalization within five weeks after the COVID-19 disease onset. *p< 0.05: Significant value.							

**Table:** 

**Table 3.** Pre-existing long-term medications used by COVID-19 patients
**Drugs n %**
**Acetylsalicylic acids** (N= 254) 37 14.6 **Proton pump inhibitors** (N= 254) 57 22.4 **Analgesics** (N= 254) 69 27.2 **Anticoagulants** (N= 254) 15 5.9 **Antihypertensives** (N= 254) 75 29.5 ACE inhibitors (N= 75) 39 52.0 Beta blockers (N= 75) 32 42.7 Calcium channel blockers (N= 75) 28 37.3 Thiazides (N= 75) 17 22.7 Angiotensin-2 receptor blockers (N= 75) 14 18.7 **Antidiabetics** (N= 254) 43 16.9 Insulin (N= 43) 9 20.9 Oral antidiabetics (N= 43) 41 95.3 Gliclazide (N= 41) 3 7.3 Vildagliptin/Linagliptin/Sitagliptin (N= 41) 6 14.6 Empagliflozin (N= 41) 1 2.4 Acarbose (N= 41) 1 2.4 Glimepiride (N= 41) 1 2.4 **Antirheumatic drugs** (N= 254) 5 2.0 **Antiarrhythmics** (N= 254) 21 8.3 **Antidepressants** (N= 254) 24 9.4 **Bronchodilators** (N= 254) 38 15.0 **Antiepileptics** (N= 254) 11 4.3 Pregabalin (N= 11) 0 0.0 Gabapentin (N= 11) 0 0.0 **Cholesterol drugs** (N= 254) 23 9.1 **Other drugs** (N= 254) 17 6.7 Alfa-1 receptor blockers (N= 17) 1 5.9 Sulfasalazine (N= 17) 1 5.9 Antihistamines (N= 17) 0 0.0 Antipsychotic drugs (N= 17) 2 11.8 Diuretics (N= 17) 4 23.5 Furosemide (N= 4) 0 0.0 Indapamide (N= 4) 3 75.0 Levothyroxine/Euthyrox (N= 17) 6 35.3 Parenteral iron (N= 17) 1 5.9 Immunosuppressives (N= 17) 1 5.9 Mycophenolate (N= 1) 1 100.0 Immunomodulatory drug (N= 17) 1 5.9
Note: Medications have multiple options, N: Total patient population, n: Number of patients using the drugs, %: Percentage of patients using the drugs.

**Table:** 

**Table 4.** Comparison of symptom count in the chronic post-COVID phase based on the five most commonly used long-term medications				
**Drugs**	**Symptom count**	**n (%)**	**Mean ± SD**	**p**
	No	179 (70.5)	2.41 ± 3.77	
**Antihypertensives**				0.49
	Yes	75 (29.5)	3.65 ± 5.48	
	No	36 (48.0)	3.94 ± 5.61	
ACE inhibitors				0.96
	Yes	39 (52.0)	3.38 ± 5.41	
	No	43 (57.3)	3.86 ± 5.53	
Beta blockers				0.86
	Yes	32 (42.7)	3.38 ± 5.48	
	No	47 (62.7)	4.09 ± 6.33	
Calcium channel blockers				0.86
	Yes	28 (37.3)	2.93 ± 3.62	
	No	17 (22.7)	3.57 ± 5.37	
Thiazides				0.47
	Yes	58 (77.3)	3.94 ± 5.99	
	No	61 (81.3)	3.66 ± 5.54	
Angiotensin-2 receptor blockers				0.00*
	Yes	14 (18.7)	3.64 ± 5.39	
**Analgesics**	No	185 (72.8)	2.46 ± 3.96	
				0.73
	Yes	69 (27.2)	3.62 ± 5.26	
	No	211 (83.1)	2.59 ± 4.17	
**Antidiabetics**				0.22
	Yes	43 (16.9)	3.67 ± 5.20	
	No	216 (85.0)	2.92 ± 4.12	
**Bronchodilators**				0.69
	Yes	38 (15.0)	2.75 ± 4.42	
	No	217 (85.4)	2.79 ± 4.25	
**Acetylsalicylic acids**				0.71
	Yes	37 (14.6)	2.68 ± 5.08	
ACE: Angiotensin-converting enzyme, n: Number of patients, (%): In parentheses, percentage of patients, SD: Standard deviation, p: Associated sample two-way analysis of variance. *p< 0.05: Significant value.

In this study, 137 female and 117 male patients with CPCS were examined, and the female sex was observed as a risk factor for developing CPCS, similar to many previous studies (23-25). However, some studies have reported that males have about the same odds of developing CPCS as females (26,27). A key factor leading to female dominance in CPCS may be sex-specific differences in immune response. Female immunological characteristics make them more sensitive to specific immune-related disease outcomes, even if males are more prone to most viral infections. Sex steroids, as well as the sex chromosome complement and related genes, are significant mediators in the formation of sex differences in immunity to viral infections (28). Regarding COVID- 19, female cells express more type I IFN signaling, T cell-associated genes, and other innate immune responses, whereas male cells express more inflammatory genes (29).

Previous studies have reported that hospitalization during acute infection is a risk factor for persistent symptoms (21-24). The most important predisposing factor for post-COVID symptoms is the severity of the disease, resulting in hospitalization. This outcome is inevitable as the patients suffer from psychological and physiological problems during prolonged hospitalizations due to severe illness (30,31). Controversially, hospitalization had no influence on CPCS in the present study. This result may be attributed to the fact that the number of non- hospitalized patients in the study sample was approximately 2.5 times higher than that of hospitalized patients. Therefore, we recommend multicenter studies that will provide statistical significance on this issue.

Positive PCR testing result was not a significant risk factor for the CPCS development in the present study. In a previous study, while only taste/smell abnormalities were higher in PCR-positive patients, there was no significant difference between PCR test positivity and the prevalence of symptoms (32). The presence of specific radiological findings on thoracic CT predicted the developing CPCS symptoms in this study-a guideline recommended that patients should be checked with chest radiography in the twelfth week after discharge. However, face-to-face outpatient control should be performed primarily for those with radiological findings and severe disease (33). The significant correlation between the specific radiological findings and persistent symptom development found as an outcome of the study supports this recommendation.

In order of frequency, comorbidities associated with persistent symptoms in COVID-19 patients were reported as follows in previous literature: 35% hypertension (HT), 16% DM, 16% cardiovascular, and 9% pulmonary (34,35). However, no association between comorbidities and CPCS was found in the study. This result might be due to the small size of the study population.

Cardiovascular disorders, including hypertension and hypertrophic cardiomyopathy, are treated with ARBs. Angiotensin-2 converting enzyme (ACE2) receptor is extensively expressed in human vascular endothelial cells, arterial smooth muscle cells, lung tissue, and gastrointestinal tract. SARS-CoV-2 enters the host cell by attaching to this receptor by the virus’s spike proteins in vivo (36). The renin-angiotensin- aldosterone system (RAAS), which controls blood pressure and is a critical factor in severe acute lung injury, can be inhibited by ACE2 receptor antagonists such as angiotensin-1 converting enzyme inhibitors (ACEIs) and ARBs (37). Therefore, some scientists have expressed concerns that RAAS inhibitors may increase the risk of COVID-19 infection and potentially lead to a poor prognosis (38). However, on April 12, 2020, the European Society of Hypertension COVID-19 Task Force declared that current evidence did not support the idea that RAAS inhibitors worsen the prognosis of COVID-19 patients (39). Moreover, in a systematic review, among patients with hypertension, the use of an ACEI/ARB was associated with lower severity of COVID-19 (OR= 0.73, 95% CI= 0.51-1.03) and lower mortality

(OR= 0.57, 95% CI= 0.37-0.87), without evidence of an increased risk of COVID-19 infection (OR= 1.00) (40). Supporting this evidence, we observed that the long-term use of ARBs had an improving effect on the chronic post-COVID symptom burden.

## Limitations

The study has several limitations. The sample size became smaller due to the patients who could not be accessed by phone and the patients who refused to participate in the study. During the study period, COVID-19 symptoms of different organ systems informed in the literature were included in the study, but various new COVID-19 symptoms have been reported in the literature until today. Our symptom list was limited to the declared symptoms at the time it was created.

## CONCLUSION

The present study showed that acute and chronic post-COVID syndromes developed significantly in COVID-19. However, the count of symptoms in the CPC phase was found to be lower compared with the APC phase. Since we encountered so many symptoms of different systems in the post-COVID phase, we recommend that patients with COVID-19 should be examined face-to-face in the APC and CPC phases.

Female sex and specific radiological findings were observed as significant risk factors for CPCS. Another important outcome of this study was the long-term use of ARBs having a positive impact on the CPCS burden. However, there is a need for multicenter studies with larger samples and diverse, multi-ethnic populations to investigate predictors of CPCS.

## Acknowledgements

We would like to thank to the healthcare professionals who work devotedly for the fight against COVID-19 in the reference chest diseases center.

**Ethical Committee Approval:** This study was approved by the İstanbul Yedikule Chest Diseases and Thoracic Surgery Training and Research Hospital Clinical Research Ethics Committee (Desicion no: 2021-119, Date: 06.05.2021).

## CONFLICT of INTEREST

The authors declare that they have no conflict of interest.

## AUTHORSHIP CONTRIBUTIONS

Concept/Design: HA, DD Analysis/Interpretation: All of authors Data acqusition: All of authors Writing: HA

Clinical Revision: HA

Final Approval: All of authors

## REFERENCES

1. *
  Johns Hopkins Coronavirus Resource Center. COVID-19 Map. Available from: https://coronavirus.jhu.edu/map. html (Accessed date: 24.07.2022).
  *
2. *
  Wiersinga WJ, Rhodes A, Cheng AC, Peacock SJ, Prescott HC. Pathophysiology, transmission, diagnosis, and treat- ment of Coronavirus disease 2019 (COVID-19): A review. JAMA 2020; 324(8): 782-3. https://doi.org/10.1001/ jama.2020.12839.
  *
3. *
  Fernández-de-Las-Peñas C. Long COVID: Current defini- tion. Infection 2021; 50(1): 285-6. https://doi. org/10.1007/s15010-021-01696-5.
  *
4. *
  Chen C, Haupert SR, Zimmermann L, Shi X, Fritsche LG, Mukherjee B. Global prevalence of post COVID-19 condi- tion or long COVID: A meta-analysis and systemat- ic review. J Infect Dis 2022; 226(9): 1593-657. https://doi. org/10.1093/infdis/jiac136.
  *
5. *
  Iqbal FM, Lam K, Sounderajah V, Elkin S, Ashrafian H, Darzi A. Understanding the survivorship burden of long COVID. EClinicalMedicine 2021; 33: 100767. https://doi. org/10.1016/j.eclinm.2021.100767.
  *
6. *
  Moreno-Pérez O, Merino E, Leon-Ramirez JM, Andres M, Ramos JM, Juan Arenas-Jiménez, et al. COVID19-ALC research post-acute COVID-19 syndrome. Incidence and risk factors: A Mediterranean cohort study. J Infect 2021; 82(3): 378-83. htpps://doi.org/10.1016/j.jinf.2021.01.004.
  *
7. *
  Anonymous Long COVID: Let patients help define long-lasting COVID symptoms. Nature 2020; 586(7828): 170. https://doi.org/10.1038/d41586-020-02796-2.
  *
8. *
  National Institute for Health and Care Excellence. COVID-
  *

*
19 rapid guideline: Managing the long-term effects of COVID-19. NICE guideline (NG188). Available from: https://www.nice.org.uk/guidance/ng188 (Accessed date: 29.01.2021).
*

9. *
  Townsend L, Dyer AH, Jones K, Dunne J, Mooney A, Gafneyy F, et al. Persistent fatigue following SARS-CoV-2 infection is common and independent of severity of initial infection. PLoS One 2020; 15(11): e0240784. https://doi. org/10.1371/journal.pone.0240784.
  *
10. *
  Halpin SJ, McIvor C, Whyatt G, Adams A, Harvey O, McLean L, et al. Postdischarge symptoms and rehabilita- tion needs in survivors of COVID-19 infection: A cross-sec- tional evaluation. J Med Virol 2020; 93(2): 1013-22. https://doi.org/10.1002/jmv.26368.
  *
11. *
  Asghar F, Shamim N, Farooque U, Sheikh H, Aqeel R. Telogen effluvium: A review of the literature. Cureus 2020; 12(5): e8320. https://doi.org/10.7759/cureus.8320.
  *
12. *
  Rizzetto G, Diotallevi F, Campanati A, Radi G, Bianchelli T, Molinelli E, et al. Telogen effluvium related to post severe SARS-CoV-2 infection: Clinical aspects and our manage- ment experience. Dermatol Ther 2020; 34(1): e14547. https://doi.org/10.1111/dth.14547.
  *
13. *
  Mieczkowska K, Deutsch A, Borok J, Guzman AK, Fruchter R, Patel P, et al. Telogen effluvium: A sequela of COVID-
  *

*
19. Int J Dermatol 2021; 60(1): 122-4. htpps://doi. org/10.1111/ijd.15313.
*

14. *
  Aksoy H, Yıldırım UM, Ergen P, Gürel MS. COVID-19 induced telogen effluvium. Dermatol Ther 2021; 34(6): e15175. https://doi.org/10.1111/dth.15175.
  *
15. *
  Iqbal FM, Lam K, Sounderajah V, Clarke JM, Ashrafian H, Darzi A. Characteristics and predictors of acute and chronic post-COVID syndrome: A systematic review and meta-analysis. EClinicalMedicine 2021; 36: 100899. https://doi.org/10.1016/j.eclinm.2021.100899.
  *
16. *
  Peçanha T, Goessler KF, Roschel H, Gualano B. Social isolation during the COVID-19 pandemic can increase physical inactivity and the global burden of cardiovascular disease. Am J Physiol Heart Circ Physio 2020; 318(6): H1441-H1446. https://doi.org/10.1152/ ajpheart.00268.2020.
  *
17. *
  Smith L, Jacob L, Yakkundi A, McDermott D, Armstrong NC, Barnett TY, et al. Correlates of symptoms of anxiety and depression and mental wellbeing associated with COVID-19: A cross-sectional study of UK-based respon- dents. Psychiatry Res 2020; 291: 113138. https://doi. org/10.1016/j.psychres.2020.113138.
  *
18. *
  Wernhart S, Weihe E, Rassaf T. Reduced physical activity and weight gain are associated with an increase of depres- sive symptoms during the COVID-19 pandemic. A general practitioners’ prospective observational study. JRSM Cardiovasc Dis 2021; 10: 20480040211047742. https:// doi.org/10.1177/20480040211047742.
  *
19. *
  Kaggwa MM, Favina A, Najjuka SM, Zeba Z, Mamun MA, Bongomin F. Excessive eating and weight gain: A rare post- acute COVID-19 syndrome. Diabetes Metab Syndr 2021; 15(5): 102252. https://doi.org/10.1016/j. dsx.2021.102252.
  *
20. *
  Messin L, Puyraveau M, Benabdallah Y, Lepiller Q, Gendrin V, Zayet S, et al. COVEVOL: Natural evolution at 6 months of COVID-19. Viruses 2021; 13(11): 2151. https://doi. org/10.3390/v13112151.
  *
21. *
  Carvalho-Schneider C, Laurent E, Lemaignen A, Beaufils E, Bourbao-Tournois C, Laribi S, et al. Follow-up of adults with noncritical COVID-19 two months after symptom onset. Clin Microbiol Infect 2020; 27(2): 258-63. https://doi. org/10.1016/j.cmi.2020.09.052.
  *
22. *
  D’Cruz RF, Waller MD, Perrin F, Periselneris J, Norton S, Smith LJ, et al. Chest radiography is a poor predictor of respiratory symptoms and functional impairment in survi- vors of severe COVID-19 pneumonia. ERJ Open Res 2021; 7(1): 00655-2020. https://doi. org/10.1183/23120541.00655-2020.
  *
23. *
  Staffolani S, Iencinella V, Cimatti M, Tavio M. Long COVID- 19 syndrome as a fourth phase of SARS-CoV-2 infection. Infez Med 2021; 30(1): 22-9. https://doi.org/10.53854/ liim-3001-3.
  *
24. *
  Bai F, Tomasoni D, Falcinella C, Barbanotti D, Castoldi R, Giovanni Mulè, et al. Female gender is associated with long COVID syndrome: A prospective cohort study. Clin Microbiol Infect 2022; 28(4): 611.e9-611.e16. https:// doi.org/10.1016/j.cmi.2021.11.002.
  *
25. *
  Sigfrid L, Drake TM, Pauley E, Jesudason EC, Olliaro P, Lim WS, et al. Long COVID in adults discharged from UK hos- pitals after COVID-19: A prospective, multicentre cohort study using the ISARIC WHO Clinical Characterisation Protocol. Lancet Reg Health Eur 2021; 8: 100186. https:// doi.org/10.1101/2021.03.18.21253888.
  *
26. *
  Moreno-Perez O, Merino E, Leon-Ramirez JM, Andres M, Ramos JM, Arenas-Jiménez J, et al. Post-acute COVID-19 syndrome. Incidence and risk factors: A Mediterranean cohort study. J Infect 2021; 82(3): 378-83. https://doi. org/10.1016/j.jinf.2021.01.004.
  *
27. *
  Stavem K, Ghanima W, Olsen MK, Gilboe HM, Einvik G. Persistent symptoms 1.5-6 months after COVID-19 in non-hospitalised subjects: A population-based cohort study. Thorax 2021; 76(4): 405. https://doi.org/10.1136/ thoraxjnl-2020-216377.
  *
28. *
  Jacobsen H, Klein SL. Sex differences in immunity to viral infections. Front Immunol 2021; 12: 720952. https://doi. org/10.3389/fimmu.2021.720952.
  *
29. *
  Liu T, Balzano-Nogueira L, Lleo A, Conesa A. Transcriptional differences for COVID-19 disease map genes between males and females indicate a different basal immunophe- notype relevant to the disease. Genes (Basel) 2020 11(12): E1447. https://doi.org/10.3390/genes11121447.
  *
30. *
  Tenforde MW, Kim SS, Lindsell CJ, Rose EB, Shapiro NI, Files DC, et al. Symptom duration and risk factors for delayed return to usual health among outpatients with COVID-19 in a multistate health care systems net- work-United States, March-June 2020. MMWR Morb Mortal Wkly Rep 2020; 69(30): 993-8. https://doi. org/10.15585/mmwr.mm6930e1.
  *
31. *
  Raveendran AV, Jayadevan R, Sashidharan S. Long COVID: An overview. Diabetes Metab Syndr 2021; 15(3): 869- 75. https://doi.org/10.1016/j.dsx.2021.04.007.
  *
32. *
  Ozcan E, Yavuzer S, Uysal BB, Islamoglu MS, Ikitimur H, Unal OF, et al. The relationship between positivity for COVID-19 RT-PCR and symptoms, clinical findings, and mortality in Turkey. Expert Rev Mol Diagn 2021; 21(2): 245-50. https://doi.org/10.1080/14737159.2021.18823 05.
  *
33. *
  British Thoracic Society. British Thoracic Society guidance on respiratory follow up of patients with a clinico-radio- logical diagnosis of COVID-19 pneumonia. Available from: www.brit-thoracic.org.uk/document-library/quality- improvement/COVID-19/resp-follow-up-guidance-post- COVID-pneumonia (Accessed date: 06.07.2020).
  *
34. *
  Huang C, Huang L, Wang Y, Li X, Ren L, Gu X, et al. 6-month consequences of COVID-19 in patients dis- charged from hospital: A cohort study. Lancet 2021; 397(10270): 220-32. https://doi.org/10.1016/S0140- 6736(20)32656-8.
  *
35. *
  Carfi A, Bernabei R, Landi F; for the Gemelli against COVID-19 post-acute care study group (2020). Persistent symptoms in patients after acute COVID-19. JAMA 2020; 324(6):603-5.https://doi.org/10.1001/jama.2020.12603.
  *
36. *
  Hoffmann M, Kleine-Weber H, Schroeder S, Krüger N, Herrler T, Erichsen S, et al. SARS-CoV-2 cell entry depends on ACE2 and TMPRSS2 and is blocked by a clinically proven protease inhibitor. Cell 2020; 181(2): 271-80.e8. https://doi.org/10.1016/j.cell.2020.02.052.
  *
37. *
  Romero CA, Orias M, Weir MR. Novel RAAS agonists and antagonists: Clinical applications and controversies. Nat Rev Endocrinol 2015; 11(4): 242-52. https://doi. org/10.1038/nrendo.2015.6.
  *
38. *
  Garami AR. Re: Preventing a COVID-19 pandemic–is there a magic bullet to save COVID-19 patients? We can give it a try. BMJ 2020; 368: m810. https://doi. org/10.1136/bmj.m810.
  *
39. *
  Kreutz R, Algharably EAE, Azizi M, Dobrowolski P, Guzik T, Januszewicz A, et al. Hypertension, the renin-angioten- sin system, and the risk of lower respiratory tract infections and lung injury: Implications for COVID-19. Cardiovasc Res 2020; 116(10): 1688-99. https://doi.org/10.1093/ cvr/cvaa097.
  *
40. *
  Liu X, Long C, Xiong Q, Chen C, Ma J, Su Y, et al. Association of angiotensin converting enzyme inhibitors and angiotensin II receptor blockers with risk of COVID- 19, inflammation level, severity, and death in patients with COVID-19: A rapid systematic review and meta-analysis. Clin Cardiol 2020; 10.1002/clc.23421. https://doi. org/10.1002/clc.23421.
  *

